# Mental health problems both precede and follow bullying among adolescents and the effects differ by gender: a cross-lagged panel analysis of school-based longitudinal data in Vietnam

**DOI:** 10.1186/s13033-019-0291-x

**Published:** 2019-05-18

**Authors:** Ha Thi Hai Le, Nam Tran, Marilyn A. Campbell, Michelle L. Gatton, Huong Thanh Nguyen, Michael P. Dunne

**Affiliations:** 1 Faculty of Social Sciences and Health Education, Hanoi University of Public Health, Hanoi, Vietnam; 2 ARC Centre of Excellence for Children and Families Over the Life Course | Institute for Social Science Research, The University of Queensland, Brisbane, Australia; 3 Faculty of Education, Queensland University of Technology, Brisbane, Australia; 4 School of Public Health and Social Work, Queensland University of Technology, Brisbane, Australia; 5 Institute for Community Health Research, Hue University, Hue, Vietnam

**Keywords:** Bullying, Victimisation, Mental health, Depression, Psychological distress, Adolescents, Reciprocal association, Cross-lagged analysis, Vietnam

## Abstract

**Background:**

The significant psychosocial harms from bullying among adolescents create major challenges for mental health promotion programs and services in schools. While the negative consequences of bullying victimisation are well known, to date there is scarce empirical analysis of inverse associations, in which mental health problems make children more vulnerable to bullying victimisation and perpetration. Based on a short-term longitudinal study among adolescents in Vietnam, this study examined reciprocal associations between children’s depressive symptoms, psychological distress, suicidal ideation and bullying victimisation experiences (i.e., victims or bully-victims).

**Methods:**

Secondary and high school students (n = 1167; age range: 11–16 years old; 55% female) in urban areas in northern Vietnam completed two self-administered questionnaires, 6-months apart in the academic year 2014–2015. Measures estimated bullying victimisation and perpetration in the past 6 months, depressive symptoms, psychological distress, and suicidal ideation. A cross-lagged analysis was performed to test the reciprocal associations.

**Results:**

About one-third of students in the sample were involved as victims, bullies or bully-victims at both times, with more males than females reporting these experiences. Females reported a higher level of depressive symptoms than males at Time 1 but not at Time 2. After adjusting for outcome variables and other covariates measured at Time 1, nine of 12 cross-lagged associations across three models were statistically significant, with different patterns for females and males. There were reciprocal associations between bullying victimisation and mental health problems. Bullying victimisation was shown as an independent predictor of subsequent mental health problems; in turn, mental health problems preceded students’ experience of becoming victims or bully-victims. Females with mental health problems were more likely to be victims; whereas similarly distressed males were vulnerable to both being bullied and being perpetrators.

**Conclusion:**

This study is the first of its kind in Vietnam and in the Southeast Asian region to examine reciprocal associations between bullying victimisation and mental health problems among adolescents. Anti-bullying intervention and prevention programs and school-based mental health promotion programs should be integrated and be sensitive to gender differences in order to maximise their impact.

**Electronic supplementary material:**

The online version of this article (10.1186/s13033-019-0291-x) contains supplementary material, which is available to authorized users.

## Background

Bullying is defined as intentional and repeated aggression that is expressed in physical, verbal, or relational forms in which the targets cannot defend themselves because of an imbalance of power [1, 2] in both traditional (i.e., face-to-face) and cyberbullying forms. Bullying is a common experience among adolescents [3–6] and strong, negative associations between bullying (including traditional and cyberbullying) and psychological wellbeing are evident among victims and perpetrators, and among children who are both victims and bullies [7–10]. Worldwide, there is a vast amount of evidence linking bullying experiences to development of emotional, cognitive, social, and behavioural problems [11–15]. The significant psychosocial harms from bullying among adolescents create major challenges for mental health promotion programs and services in schools.

While the negative consequences of bullying victimisation are well known, to date few longitudinal studies have investigated an inverse association, in which mental health problems make individuals more vulnerable to becoming victims, bullies, or both [16–20]. For example, a 3-year longitudinal study in Australia among 1504 adolescents aged 13-years at baseline found students who had social and emotional difficulties were more likely to be victims of both cyberbullying and traditionally bullying [18]. The Australian study results are in line with a study conducted among adolescents in the United States which also reported children with emotional, developmental, and behavioural problems were more likely to experience bullying victimisation [21]. A South Korean study with students aged 10- to 13-years old found depression at baseline was significantly associated with later traditional and cyberbullying victimisation and perpetration, and high anxiety was associated with perpetration of cyberbullying [17]. The relationship between mental health problems and bullying is complex as studies have assessed different aspects of mental health, such as social and emotional difficulties [18, 21], narcissism, self-esteem, impulsivity [20, 22, 23], depressive symptoms and anxiety [17]. Not all studies agree with one study finding that psychopathologic behaviour is a consequence rather than a cause of a bullying victimisation experience [19] Other studies have found no gender differences in such associations [20], while some have revealed a variation between males and females in this regard [16, 23].

To date, there has been very little relevant research in Vietnam and Southeast Asian countries examining the inverse association; and whether this is similar for males and females. Based on a short-term longitudinal study among adolescents aged 11- to 16-years in Vietnam, this study aimed to provide empirical evidence linking depression, psychological distress, and suicidal ideation to subsequent bullying victimisation, and to examine whether the reciprocal association differs between males and females.

## Method

### Participants and procedure

We analysed longitudinal data from a survey at two-time points, 6-months apart, at four urban, public, middle schools (including students aged 11–13) and high schools (students aged 15–16) in the Red River Delta in Vietnam during the academic year 2014–2015. An identity number matching technique that enables anonymous matching of individuals across surveys was employed [24]. The surveys were conducted during non-teaching sessions, using self-administered questionnaires which took respondents approximately 45 min to complete. Of 1539 students recruited from 29 classes who participated in a baseline survey (Time 1), 1460 (94.9%) students were followed up 6 months later (Time 2). Further details of the survey sampling have been described elsewhere [10]. The final sample for analyses in this study included 1167 students (82.0%) who provided full information across variables of interest at both time points. These students are those who reported as victims only, bully-victims, and non-involvement in any form of bullying. Bullying perpetrators are not included as they accounted for a small number of population in this analysis.

The study was approved by the Human Research Ethics Committees of the Queensland University of Technology (Australia) (No. 1400000713) and the Hanoi School of Public Health (Vietnam) (No. 279/2014/YTCC-HD3). Informed consent was obtained from the principals of the four participating schools and from all individual participants included in the study.

### Measures

#### Measure of bullying involvement

To measure bullying victimisation, we included six behaviours used in previous studies [1, 25, 26] to assess adolescents’ involvement in traditional and cyberbullying victimisation and perpetration: (i) hitting/kicking/shoving around, (ii) robbing/stealing/damaging properties, (iii) threatening/forcing someone to do things they do not want to do, (iv) using mean names/teasing in rude ways, (v) excluding, and (vi) spreading rumours. Before answering the questionnaire, students were given an adapted definition of bullying [2, 27] to help them understand bullying. The scale has been validated among Vietnamese students through a pilot phase of this study and published elsewhere [28]. For the victimisation scale, students were asked, ‘How often have you been bullied in any way during the last 6 months?’, then six responses were presented. The bullying perpetration measurement was similar, with questions about how often they bullied others. We distinguished traditional bullying from cyberbullying via the different modes (in-person or cyber) in which students experienced bullying behaviours. A five-point Likert scale, ranging from 0 = never, 1 = a few times during the last 6 months, 2 = once or twice a month, 3 = once or twice a week, 4 = almost every day, was used to measure frequency of behaviour, for each mode of bullying. In this study, experiencing such behaviours once or twice a month (cut-off point = 2) was selected to measure bully victimisation and perpetration. This cut-off point has been commonly used in prior studies [29, 30]. Similar to previous studies [9, 31], bullying victimisation was categorised into two distinct groups: those who are victimised (victims), and those who are victimised and simultaneously bullied others (bully-victim).

#### Measures of mental health problems

The study assessed mental health problems consisting of depressive symptoms, psychological distress, and suicidal ideation. First, depressive symptoms were measured using the *Centre for Epidemiological Studies*-*Depression Scale* (CES-D) [32]. The scale comprises 20 items (e.g., I felt lonely) using a four-point Likert scale. Respondents were asked to indicate the frequency with which they had experienced each feeling during the previous week with the following response options: 0 = less than 1 day, 1 = 1–2 days, 2 = 3–4 days, and 3 = 5–7 days. Total scores were calculated by summing responses across the 20 items creating a total, ranging from zero to 60, with the higher scores indicating higher levels of depressive symptoms. The scale was validated among Vietnamese students [33]. Alpha coefficients for the scale were 0.86 and 0.87 for Times 1 and 2, respectively in the present study.

Psychological distress was assessed using the *Kessler Psychological Distress Scale* (K10) [34], which has been used in prior studies among Vietnamese school adolescents [35, 36]. The scale includes 10 items (e.g., During the last 30 days, about how often did you feel tired out for no good reason?) to measure emotional feelings experienced in the last month using a five-point Likert scale ranging from ‘1 = none of the time’ to ‘5 = all of the time’. A composite score was generated with a higher value indicating higher levels of psychological distress. Alpha coefficients for the K10 were 0.87 and 0.92 for Times 1 and 2, respectively for this sample.

We measured suicidal ideation using three items adapted from the American School Health Association Survey [37]. Respondents were asked, ‘During the past 6 months, have you ever (i) seriously thought about attempting suicide? (ii) made a specific plan about how you would attempt suicide? and (iii) attempted suicide?’. The responses were categorised as a dichotomous variable with 0 = no and 1 = yes if respondents admitted at least one of these thoughts or behaviours. The scale has been previously employed in other studies conducted among Vietnamese adolescents with excellent psychometric properties [35, 36, 38].

#### Measures of covariates

Demographic characteristics comprised gender (female vs male), age in years, and family structure (living with both parents, living with one parent, living apart due to separation, divorce). Other information such as witnessing parental violence, conflict with siblings, perceiving other students and teachers as helping stop bullying, spending time online with technology devices, and receiving support from family and friends were selected characteristics based on the literature [36, 38, 39].

Consistent with previous studies [36, 38], we assessed whether students had witnessed parents seriously arguing or fighting by asking them, ‘How often have you witnessed your parents having (i) a serious argument with each other? and (ii) physically fighting with each other?’. Possible answers were on a four-point Likert scale ranged from ‘1 = never’ to ‘4 = often’. Alpha coefficients were 0.71 and 0.74 at Time 1 and 2, respectively for this sample.

Conflict with siblings was assessed by one question: ‘How often have you had serious conflict (argument, fighting, etc.) with your siblings?’ Response options were on a four-point Likert scale, ranging from ‘1 = no sibling/never’ to ‘4 = often’. Scores that fell below the mean were coded ‘0 = infrequent’ and those that fell above the mean were coded as ‘1 = frequent’. The question has been used in previous studies of adolescents in Vietnam [36, 38].

Perceptions of friends and teachers attempts to stop bullying at school were assessed by asking students, ‘How often do (i) teachers/other adults try to stop it when a student is being bullied at school? and (ii) students at school try to stop it when a student is being bullied at school?’; using a five-point Likert scale ranging from ‘1 = almost never’ to ‘5 = almost always’ [25]. Scores were dichotomised for data interpretation purposes with the cut-off point of 3 or above signifying sometimes/often and scores of 2 or below signifying almost never.

Online activities were measured with four items that asked respondents about time spent in the past week in online activities, including communication, social networking, entertainment, and other activities. The five-point Likert scale response options were 1 = never use, 2 = several times a week, 3 = several times a day, 4 = several times an hour, 5 = all the time. These were summed with scores ranging from 4 to 20; a higher score indicates higher time spent on online activities. Alpha coefficients for this scale in this sample were α = 0.60 and α = 0.64 for Time 1 and 2, respectively.

Social support from family and friends was measured by employing the *Multidimensional Scale of Perceived Social Support* (MSPSS) [39]. The MSPSS comprises a 12-item scale equally distributed to measure family support (e.g., My family really tries to help me) and friend support (e.g., My friends really try to help me) with response options on a four-point Likert scale ranging from ‘1 = strongly disagree’ to ‘4 = strongly agree’. The response scores were summed, with a higher total score indicating higher levels of support. Alpha coefficients for these subscales at Time 1 and Time 2, respectively, were 0.88 and 0.89 for family support and 0.91 and 0.93 for friend support in this sample.

### Analyses

**Fig. 1:**
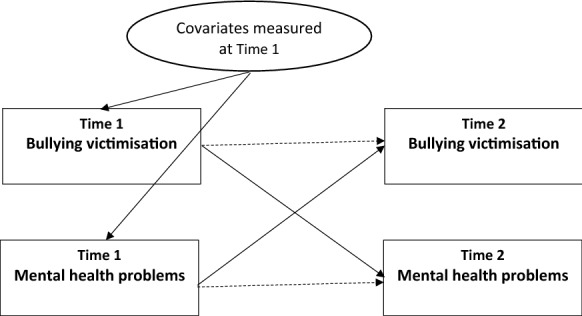
GSEM model measures cross-lagged relationship between bullying victimisation and mental health problems

A cross-lagged panel analysis [40, 41] was employed to conceptualise and test the possibility that mental health problems and bullying victimisation are reciprocally related over time, while statistically controlling for the value of the outcome variable and covariates measured at Time 1. The present study hypothesised reciprocal relationships between mental health problems (as measured by depressive symptoms, psychological distress, and suicidal ideation) and bullying victimisation (victims and bully-victims). Specifically, we examined the effects of Time 1 (baseline) mental health problems on Time 2 bullying victimisation and the effects of Time 1 bullying victimisation on Time 2 mental health problems (Fig. 1).

The data analyses were performed in three stages with Stata/SE 15.0 for Windows. First, *Chi square* and *t test* analyses were used to examine group difference between male and female students for all variables. At the second stage, as the key outcomes of interest are both categorical (bullying victimisation and suicidal ideation) and continuous variables (depressive symptom and psychological distress), generalised structural equation modelling (GSEM) in Stata/SE 15.0 was used with linear regression, logit regression, and multinomial logit regression to estimate cross-lagged path models. GSEM is a statistical modelling technique employed to analyse structural relationships between multiple variables where responses or outcome variables are continuous, binary, and multinomial with various regressions as appropriate [42]. Our first model estimated the lagged effects of bullying victimisation and the symptoms of depression and vice versa. For the second model, we examined the reciprocal association between bullying victimisation and psychological distress. For the last model, we investigated the bi-directionality of the association between bullying victimisation and suicidal ideation. We included covariates (as described above) in each model as GSEM and cross-lagged panel data analyses can incorporate previous levels of key variables in the analyses [40]. In addition, we compared log likelihoods from nested models (with and without covariates) to determine the impact on model fit (data not shown). The group option function of GSEM was employed to fit the model for two groups—female and male students. In this study, GSEMs are estimated using multinomial, Bernoulli, and Gaussian family with logit and identity link to investigate such reciprocal associations. As a result, data presented in the result section are reported for relative risk ratio (RRs), odds ratio (OR), and coefficient (Coef.), respectively.

Test–retest reliability over 6 months was calculated to examine how it might influence the study findings. Intra-cluster correlation coefficient (ICC) estimates and their 95% confident intervals were calculated for depressive symptoms and psychological distress based on mean-rating (k = 2), absolute-agreement, 2-way mixed-effects model. The Cohen’s Kappa statistic was conducted for suicidal ideation and bullying involvement.

Finally, among the 18% of students excluded from our analyses, about 6% (70 students) were categorised as perpetrators only. It is noted that our study focused only on students who reported as victims only or bully-victims in comparison with those who reported not being involved in any form of bullying. The prevalence of bullying perpetration alone was too low for further analysis. To assess the impact of missing data we conducted multivariate logistic regression to compare the profile of students who had complete data at Time 1 and Time 2 who were included in the present analyses with those students who had been excluded from the analyses because of missing data.

## Results

In the overall sample, female students accounted for nearly 55% with an average age of 13.92 years (SD = 1.86) compared with 13.45 years (SD = 1.89) for their male counterparts (Table 1). Almost 88% of students were living with both parents at Time 1 while the others reported they were living with a single parent because of parental separation or divorce. About one-third of students at Time 1 and one-quarter of students at Time 2 were involved in bullying. The prevalence of bullying involvement at Time 1 was higher than at Time 2 (*p* < 0.05). Male students experienced bullying in any form more than the female students (*p *< 0.05). More females than males experienced depressive symptoms at Time 1 (p < 0.05) but not at Time 2. The likelihood of suicidal ideation among females was slightly higher than among males in both surveys, although the differences were not statistically significant (Table 1).

**Table 1:** Descriptive statistics of key variables across times by gender

Variables	Full sample		Female		Male		p value
	N	Mean (SD) or %	N	Mean (SD) or %	N	Mean (SD) or %	
Age at Time 1	1424	13.71 (1.89)	782	13.92 (1.86)	642	13.45 (1.89)	0.000
Sibling conflict	1424	2.24 (1.01)	782	2.36 (1.00)	642	2.09 (1.0)	0.000
Witness to parental violence	1424	3.11 (1.30)	782	3.19 (1.30)	642	3.02 (1.28)	0.011
Family structure							
Living together	1221	87.78	668	87.32	553	88.34	0.564
Others (separated, divorced)	170	12.22	97	12.68	73	11.66	
Bullying victimisation at Time 1							
Not involved	867	60.88	521	66.62	346	53.89	
Victim	373	26.19	169	21.61	204	31.78	0.000
Bully-victim	134	9.41	64	8.18	70	10.90	
Bully	50	3.51	28	3.58	22	3.43	
Bullying victimisation at Time 2							
Not involved	1127	79.14	632	80.82	495	77.10	
Victim	188	13.20	99	12.66	89	13.86	0.268
Bully-victim	76	5.34	36	4.60	40	6.23	
Bully	33	2.32	15	1.92	18	2.80	
Depressive symptoms at Time 1	1424	14.73 (8.99)	782	15.21 (9.06)	642	14.15 (8.87)	0.025
Depressive symptoms at Time 2	1424	15.10 (9.64)	782	14.91 (9.21)	642	15.34 (10.15)	0.40
Psychological distress at Time 1	1424	19.39 (7.42)	782	19.69 (7.20)	642	19.03 (7.67)	0.10
Psychological distress at Time 2	1424	19.29 (8.12)	782	19.45 (7.76)	642	19.09 (8.53)	0.40
Suicidal ideation at Time 1							
No	1220	85.67	661	84.53	559	87.07	
Yes	204	14.33	121	15.47	83	12.93	0.173
Suicidal ideation at Time 2							
No	1241	87.15	677	86.57	564	87.85	0.473
Yes	183	12.85	105	13.43	78	12.15	

### Associations between bullying victimisation and depressive symptoms

As shown in Table 2, controlling for other covariates—including symptoms of depression at Time 1—students categorised as victims or bully-victims at Time 1 reported an average of 1.39 and 2.02 more depressive symptoms at Time 2 compared to those who were not involved in any form of bullying (*p *< 0.05). There were differences by gender, with more depressive symptoms shown by males than females at Time 2 (Table 2).

**Table 2:** GSEM model investigating the reciprocal association between bulling involvement and depressive symptoms by gender

Bullying victimisation at Time 1	Depressive symptom at Time 2^a^					
	Full sample		Female		Male	
	Coef. (95% CI)	*p* value	Coef. (95% CI)	*p* value	Coef. (95% CI)	*p* value
Not involved (ref.)	1.0	–	1.0	–	1.0	–
Victims	1.39* (0.20–2.59)	0.02	− 0.59 (− 2.19–1.01)	0.47	2.87*** (1.12–4.63)	0.00
Bully-victims	2.02* (0.10–3.93)	0.04	0.33 (− 1.92–2.59)	0.77	3.22** (0.71–5.73)	0.01

Depressive symptoms at Time 1	Bullying victimisation at Time 2 (Ref: not involved)^b^					
	Full sample		Female		Male	
	RRs (95% CI)		RRs (95% CI)		RRs (95% CI)	
	Victims	Bully-victims	Victims	Bully-victims	Victims	Bully-victims
Depression	1.02* (1.00–1.04)	1.02 (0.99–1.05)	1.04** (1.01–1.07)	1.01 (0.97–1.06)	1.00 (0.97–1.03)	1.04 (1.00–1.08)

Coef., coefficient; 95% CI, 95% confident interval; RRs, relative-risk ratio

* p < 0.05, ** p < 0.01 and *** p < 0.001

^a^GSEM models were adjusted for previous depression symptoms and other covariates including age, family support, friend support, witnessing parental violence, sibling conflict, student’s time spent online, perceiving other students and teachers as helping stop bullying, and family structure

^b^GSEM models were adjusted for previous bullying victimisation and similar covariates in depression symptom models

Also shown in Table 2, the analyses suggested having a higher level of depressive symptoms at Time 1 increases the likelihood that a respondent will be involved as a bullying victim at Time 2 (*p* = 0.01). Specifically, each increase of one in the depressive symptom score at Time 1 was associated with an 1.2-fold increased risk of experiencing bullying victimisation at Time 2 (Table 2). A gender difference exists, with females more likely to be victims than males. There was no statistical association between the symptoms of depression at Time 1 and being a bully-victim at Time 2.

### Associations between bullying victimisation and psychological distress

Regarding the effects of bullying victimisation on psychological distress, there was no significant association between being a victim at Time 1 and psychological distress at Time 2 (Table 3); however, those who were bully-victims at Time 1 had significantly higher psychological distress at Time 2, compared to those not involved in bullying at Time 1 (β = 1.82, *P* < 0.05). This association was stronger for females than for males (Table 3).

**Table 3:** GSEM model investigating the reciprocal association between bulling involvement and psychological distress by gender

Bullying victimisation at Time 1	Psychological distress at Time 2^a^					
	Full sample		Female		Male	
	Coef. (95% CI)	*p* value	Coef. (95% CI)	*p* value	Coef. (95% CI)	*p* value
Not involved (ref.)	1.0	–	1.0	–	1.0	–
Victims	0.85 (− 0.12–1.82)	0.09	0.11 (− 1.15–1.38)	0.86	1.41 (− 0.08–2.90)	0.06
Bully-victims	1.82* (0.30–3.35)	0.02	1.77* (− 0.01–3.55)	0.051	1.78 (− 0.36–3.91)	0.10

Psychological distress at Time 1	Bullying victimisation at Time 2^b^ (Ref: not involved)					
	Full sample		Female		Male	
	RRs (95% CI)		RRs (95% CI)		RRs (95% CI)	
	Victims	Bully-victims	Victims	Bully-victims	Victims	Bully-victims
Psychological distress	1.03* (1.00–1.05)	1.03* (1.00–1.07)	1.05** (1.01–1.09)	1.02 (0.97–1.08)	1.01 (0.98–1.04)	1.05* (1.00–1.09)

* p < 0.05, ** p < 0.01 and *** p < 0.001

^a^GSEM models were adjusted for previous psychological distress and covariates including age, family support, friend support, witnessing parental violence, sibling conflict, student’s time spent online, perceiving other students and teachers as helping stop bullying, and family structure

^b^GSEM models were adjusted for previous bullying victimisation and similar covariates in psychological distress models

Regarding the effect of psychological distress on subsequent bullying victimisation, the adjusted multinomial logistic regression GSEM model indicated that a point increase in psychological distress at Time 1 was associated with a 3% increase in the odds of being a victim or bully-victim at Time 2 (95% CI 1.00 to 1.05 for victims; and 1.00 to 1.07 for bully-victim). An effect was found for both males and females, although there was a notable difference: psychological distress at Time 1 was significantly associated with victimisation only for females but was combined bully-victim status also for males (Table 3).

### Associations between bullying victimisation and suicidal ideation

Results from the cross-lagged analyses for bullying victimisation and suicidal ideation showed that in comparison with students who were non-victims at Time 1, those who were victims only or both bully-victims were almost twice as likely to report suicidal ideation at Time 2 (OR = 1.83, 95% CI 1.01 to 3.32 for bully-victims; and OR = 2.02, 95% CI 1.33 to 3.06 for victims). The model fitted for the male and female groups showed that females were more vulnerable than their male counterparts (Table 4).

**Table 4:** GSEM model investigating the reciprocal association between bulling involvement and suicidal ideation by gender

Bullying victimisation at Time 1	Suicidal ideation at Time 2^a^					
	Full sample		Female		Male	
	No (ref.)	Yes-OR (95% CI)	No (ref.)	Yes-OR (95% CI)	No (ref.)	Yes-OR (95% CI)
Not involved (ref.)	1.0	–	1.0	–	1.0	–
Victims	1.0	2.02*** (1.33–3.06)	1.0	2.12** (1.21–3.72)	1.0	1.88 (0.98–3.60)
Bully-victims	1.0	1.83* (1.01–3.32)	1.0	2.30* (1.07–4.92)	1.0	1.32 (0.54–3.23)

Suicidal ideation at Time 1	Bullying victimisation at Time 2^b^ (Ref: not involved)					
	Full sample		Female		Male	
	RRs (95% CI)		RRs (95% CI)		RRs (95% CI)	
	Victims	Bully-victims	Victims	Bully-victims	Victims	Bully-victims
No (ref.)	1.0	1.0	1.0	1.0	1.0	1.0
Yes	1.45 (0.91–2.31)	2.21* (1.17–4.15)	1.51 (0.81–2.82)	2.27 (0.94–5.05)	1.54 (0.71–3.34)	2.55* (1.00–6.56)

* p < 0.05, ** p < 0.01 and *** p < 0.001

^a^GSEM models were adjusted for previous suicidal ideation and covariates including age, family support, friend support, witnessing parental violence, sibling conflict, student’s time spent online, perceiving other students and teachers as helping stop bullying, and family structure

^b^GSEM models were adjusted for previous bullying victimisation and similar covariates in suicidal ideation models

Regarding the effect of suicidal ideation on subsequent bullying victimisation, the GSEM model revealed those who had suicidal ideation at baseline were 2.21 (OR = 2.21, 95% CI 1.17 to 4.15) times more likely to be in the bully-victim group at Time 2. There was no statistically significant association between suicidal ideation at Time 1 and being a victim at Time 2. Males with suicidal ideation at time 1 had significantly higher risk of being bully-victims at Time 2 (OR = 2.55, 95% CI 1.00 to 6.56). This association between suicidality and later bullying appears to be similar for females but was not statistically significant (Table 4).

## Discussion

The main purpose of this study was to investigate the reciprocal associations between mental health problems and bullying experiences. After adjusting for outcome variables and other covariates measured at Time 1, nine of 12 cross-lagged associations across three models were found to be statistically significant. There were somewhat different patterns for females and males (Additional file 1: Table S1). Overall, the relationship between bullying victimisation and mental health problems appears to be reciprocal: bullying victimisation is an independent factor predicting subsequent mental health problems, and in turn, mental health problems influence the likelihood that students become victims or bully-victims. This study is the first of its kind in Vietnam and in Southeast Asian countries to examine reciprocal associations between bullying victimisation and mental health problems among adolescents.

Our findings support previous studies among adolescents which show that being a victim or a bully-victim significantly predicted subsequent depression and suicidal ideation [14, 31, 43, 44]. However, the data are inconsistent with some observations [15, 45] regarding bullying and subsequent psychological distress. We found that bully-victims had higher psychological distress at Time 2, but surprisingly not those who were victims only. This might be due to sample size limitations or unknown covariation, but it is plausible that the impact of bullying exposure on distress is additive, and adolescents with dual involvement have more trauma than those with one role alone.

As found in previous studies [46, 47], the associations between bullying victimisation and mental health problems vary between males and females. Victimised males were more likely to have depressive symptoms and their female counterparts disclosed more suicidal ideation; moreover, female bully-victims were more likely to experience psychological distress.

This study confirmed the correlation between pre-existing mental health problems and bullying victimisation suggested in cross-sectional [48, 49] and longitudinal studies [16, 17]. The Vietnamese students with psychological distress were significantly more likely to be both victims and bully-victims; while those with depressive symptoms were more likely to be victims but not bully-victims. Further, those with suicidal ideation appear more likely to become bully-victims but not victims. Interestingly, this study found that females with mental health problems are more likely to be victims at Time 2, whereas their male counterparts with mental health problems tended to become bully-victims. This is consistent with a general tendency for young males to externalise mental distress by aggression [17, 46].

Assessment of test–retest reliability found moderate reliability between the measures administered at T1 and T2 with ICC = 0.60 and 0.65, respectively for depressive symptoms and psychological distress. For suicidal ideation and bullying involvement, the Cohen’s Kappa statistic showed high and substantial strength of agreement with k = 83.3 and 66.3, respectively. The moderate and high coefficients imply that individual differences in change over the 6-month period were small relative to the individual differences in the baseline scores.

This study makes several contributions to policy and research. First, the findings should be interpreted within the Vietnam schooling context where there is limited availability of mental health support services and few systematic programs for prevention of, and response to bullying among students. There is an urgent need to implement in practical ways the recent national agenda (Decree No. 80/2017/ND-CP in 2017) which aims to ensure safe, healthy, and friendly environments in schools, including prevention of violence [50]. Programs in schools should integrate anti-bullying efforts and mental health promotion to maximise their impact. Further, the evidence in this study clearly suggests that such programs should be sensitive to gender differences in bullying behaviours and their effects.

Second, to our knowledge, this is the first study in Vietnam and in the Southeast Asian region to illustrate reciprocal associations between bullying victimisation and mental health problems among adolescents. The analysis of cross-lagged associations between mental health problems and bullying victimisation shows complex patterns. Further research with longitudinal designs and multiple follow-ups over longer time periods may be useful to determine the strength of the findings observed here.

Another contribution to the literature is our findings regarding gender differences in the reciprocal associations. The apparent gender difference in the impact of bullying victimisation on mental health are consistent with previous literature [9, 46, 51]. The results also confirm a gender difference in the inverse association between mental health problems and bullying victimisation [23]. Further, the analysis of gender differences among Vietnamese youth shows that female students with mental health problems are more vulnerable to being victimised while male victims tend to externalise and be aggressive toward others [17, 46].

This study has several limitations. First, we measured bullying behaviours and mental health problems among school students only and did not include young people who do not attend school. Further studies in Vietnam and southeast Asian countries should be broadened to include many community settings where young people can be exposed to violence and intimidation by peers [52, 53]. Second, when examining relationships with mental health problems, we concentrated on victims and bully-victims and not perpetrators because the number of adolescents who were perpetrators only was too low for detailed analysis (about 6%). Third, we could only include confounding factors that are available in our dataset. Some uncontrolled confounding variables such as other common mental disorders or hostile-reactive parenting may have affected the findings, and hence the absence is a limitation of this study. Fourth, given that bullying behavior varies between and within groups, correlations within sample clusters, and variation between clusters should be estimated. Intention to examine cluster effects should be built into planning the study design (by surveying more clusters than was achieved here). Fifth, future studies should confirm the reciprocal associations between bullying roles and mental health problems among adolescents in a design with multiple waves and longer duration instead of just two waves 6-months apart. Missing data was also a limitation of this study. Sensitivity analysis comparing those who provided complete data with those who had missing data showed that older students who did not live with their parents, and those who perceived (at Time 1) that there was teacher support to stop bullying, were more likely to have missing data at Time 2 (Additional file 1: Table S2).

## Conclusions

This study showed that the relationships between bullying victimisation and mental health among Vietnamese adolescents appear to be reciprocal. Bullying victimisation impacts mental health problems, and in turn, mental health problems impact bullying victimisation. The evidence confirms that both bullying victimisation and mental health problems are important risk factors to be targeted in preventive interventions. Thus, to maximise the impact of antibullying programs and mental health promotion, there needs to be an integrated approach.

## Additional file

**Additional file 1: Table S1.** Summary cross-lagged associations between bullying victimisation and mental health problems among adolescents. **Table S2.** Multivariate analyses predicting those included in main analyses vs those excluded in main analyses.

## Data Availability

The dataset used and/or analysed during the current study are not available because of a confidential agreement with the respondents.

## Abbreviations

GSEM: general structural equation modelling
SD: standard deviation
Coef: coefficient
OR: odds ratio
RRs: relative risks
CI: confident interval
vs: versus
ICC: intra-cluster correlation coefficient
