# Supplementary material for: Mental health problems both precede and follow bullying among adolescents and the effects differ by gender: a cross-lagged panel analysis of school-based longitudinal data in Vietnam
Source: Int J Ment Health Syst. 2019 May 18;13:35. doi: 10.1186/s13033-019-0291-x (PMC6525446; doi:10.1186/s13033-019-0291-x)
Supplement: Supplementary file 1 — Additional file 1: Table S1. Summary cross-lagged associations between bullying victimisation and mental health problems among adolescents. Table S2. Multivariate analyses predicting those included in main analyses vs those excluded in main analyses. [file 13033_2019_291_MOESM1_ESM.docx]

ADDITIONAL MATERIAL

**Mental health problems both precede and follow bullying among adolescents and the effects differ by gender: a cross-lagged panel analysis of school-based longitudinal data in Vietnam**

Ha Thi Hai Le^1^, Nam Tran^2^, Marilyn A. Campbell^3^, Michelle L. Gatton^4^, Huong Thanh Nguyen^1^, and Michael P. Dunne^4,5*^

^1^ Faculty of Social Sciences and Health Education, Hanoi University of Public Health, Hanoi, Vietnam.

^2^ ARC Centre of Excellence for Children and Families over the Life Course | Institute for Social Science Research, The University of Queensland, Brisbane, Queensland, Australia.

^3^ Faculty of Education, Queensland University of Technology, Brisbane, Australia.

^4^ School of Public Health and Social Work, Faculty of Health, Queensland University of Technology, Brisbane, Australia.

^5^ Institute for Community Health Research, Hue University, Hue, Vietnam.

**Correspondent:*

Michael P. Dunne

Email: [m.dunne@hueuni.edu.vn](mailto:m.dunne@hueuni.edu.vn)

| **Table S1 Summary cross-lagged associations between bullying victimisation and mental health problems among adolescents** | | | |  |
| --- | --- | --- | --- | --- |
| **Reciprocal association** | **Full sample** | **Gender** | |  |
|  |  | **Female** | **Male** |  |
| Depressive symptoms – Bullying victimisation | | | | |
| Depressive symptom 🡪 victim only | + | + | - |  |
| Depressive symptom 🡪 bully-victims | - | - | - |  |
| Victim only 🡪 depressive symptom | + | - | + |  |
| Bully-victims 🡪 depressive symptom | + | - | + |  |
| Psychological distress – bullying victimisation | | | | |
| Psychological distress 🡪 victim only | + | + | - |  |
| Psychological distress 🡪 bully-victims | + | - | + |  |
| Victim only 🡪 psychological distress | - | - | - |  |
| Bully-victims 🡪 psychological distress | + | + | - |  |
| Suicidal ideation – bullying victimisation | | | | |
| Suicidal ideation 🡪 victim only | - | - | - |  |
| Suicidal ideation 🡪 bully-victims | + | - | + |  |
| Victim only 🡪 suicidal ideation | + | + | - |  |
| Bully-victims 🡪 suicidal ideation | + | + | - |  |

Note: + indicates significant association; - indicates non-significant association.

| **Table S2 Multivariate analyses predicting those included in main analyses vs those excluded in main analyses** | | |
| --- | --- | --- |
| **Predictors^1^** | **Odds of being lost to follow-up** | |
|  | **Unadjusted** | **Adjusted^2^** |
|  | *OR/Coef. (95% CI)* | *OR/Coef. (95% CI)* |
| **Sex of respondents** |  |  |
| Female (ref.) | 1.0 | 1.0 |
| Male | 1.21 (0.89 - 1.65) | 1.63 (0.97 - 2.73) |
| **Age** | *0.22 (.014 - 0.31)* | *1.26 (1.09 - 1.45)* |
| **Family structure** |  |  |
| Parents living together (ref.) | 1.0 | 1.0 |
| Others | 0.58 (0.31 - 1.06) | *0.12 (0.02 - 0.86)* |
| **Seeing parental violence** | -0.06 (-0.19 - 0.06) | -0.20 (-0.46 - 0.06) |
| **Sibling conflict** | -0.09 (-0.25 - 0.06) | -0.04 (-0.31 - 0.23) |
| **Perceived stopping of bullying by teachers** | |  |
| Almost never (ref.) | 1.0 | 1.0 |
| Sometime/Often | 0.80 (0.54 - 1.19) | *0.53 (0.29 - 0.99)* |
| **Perceived stopping of bullying by students** | |  |
| Almost never (ref.) | 1.0 | 1.0 |
| Sometime/Often | 1.25 (0.84 - 1.84) | 1.38 (0.73 - 2.61) |
| **Depressive symptoms at Time 1** | -.01 (-0.03 - 0.01) | -0.01 (-0.05 - 0.03) |
| **Psychological distress at Time 1** | *-0.03 (-0.05 - -0.003)* | -0.02 (-0.07 - 0.03) |
| **Suicidal Ideation at Time 1** |  |  |
| Yes (ref.) | 1.0 | 1.0 |
| No | *0.45 (0.25 - 0.79)* | 1.10 (0.49 - 2.49) |
| **Bullying victimisation at Time 1** |  |  |
| Not involved (ref.) | 1.0 | 1.0 |
| Victims | 1.10 (0.77 - 1.55) | 1.13 (0.64 - 1.99) |
| Bully-victims | 0.64 (0.34 - 1.19) | 0.61 (0.21 - 1.78) |
| Bullies | 0.72 (0.28 - 1.85) | 0.52 (0.07 - 4.01) |

Note: Italic = *p* <0.05;
